# Changes in the Complexity of Heart Rate Variability with Exercise Training Measured by Multiscale Entropy-Based Measurements

**DOI:** 10.3390/e20010047

**Published:** 2018-01-17

**Authors:** Frederico Sassoli Fazan, Fernanda Brognara, Rubens Fazan Junior, Luiz Otavio Murta Junior, Luiz Eduardo Virgilio Silva

**Affiliations:** 1 Department of Physiology, School of Medicine of Ribeirão Preto, University of São Paulo, Ribeirão Preto, SP 14049-900, Brazil; 2 Department of Computing and Mathematics, School of Philosophy, Sciences and Languages of Ribeirão Preto, University of São Paulo, Ribeirão Preto, SP 14040-901, Brazil; 3 Department of Computer Science, Institute of Mathematics and Computer Sciences, University of São Paulo, São Carlos, SP 13566-590, Brazil

**Keywords:** sample entropy, dispersion entropy, multiscale entropy, complexity, heart rate variability, rat, exercise, physical training, conditioning

## Abstract

Quantifying complexity from heart rate variability (HRV) series is a challenging task, and multiscale entropy (MSE), along with its variants, has been demonstrated to be one of the most robust approaches to achieve this goal. Although physical training is known to be beneficial, there is little information about the long-term complexity changes induced by the physical conditioning. The present study aimed to quantify the changes in physiological complexity elicited by physical training through multiscale entropy-based complexity measurements. Rats were subject to a protocol of medium intensity training (n=13) or a sedentary protocol (n=12). One-hour HRV series were obtained from all conscious rats five days after the experimental protocol. We estimated MSE, multiscale dispersion entropy (MDE) and multiscale SDiff${}_{q}$ from HRV series. Multiscale SDiff${}_{q}$ is a recent approach that accounts for entropy differences between a given time series and its shuffled dynamics. From SDiff${}_{q}$, three attributes (*q*-attributes) were derived, namely SDiff${}_{q_{max}}$, $q_{max}$ and $q_{zero}$. MSE, MDE and multiscale *q*-attributes presented similar profiles, except for SDiff${}_{q_{max}}$. $q_{max}$ showed significant differences between trained and sedentary groups on Time Scales 6 to 20. Results suggest that physical training increases the system complexity and that multiscale *q*-attributes provide valuable information about the physiological complexity.

## 1. Introduction

The study of system complexity is very challenging and has attracted much attention in the past few years [1,2,3]. Physiological complexity reflects the interoperability and correct functioning of regulatory processes as a whole, so the higher the complexity, the higher the system ability to adapt to different situations in daily life [4].

Heart rate variability (HRV) series, derived from the electrocardiogram (ECG) or arterial pressure signals, is one of the most important sources of information about system physiological status. Heart rate is actively controlled by the autonomic nervous system and can respond to many situations when the organism is challenged. A number of studies demonstrated that many indices extracted from HRV are powerful risk predictors of morbidity and death, for cardiac and non-cardiac diseases [5,6,7].

One of the most substantial challenges in the quantification of complexity from HRV time series is the difficulty in finding out a single measurement capable of doing this task consistently. In other words, most of the complexity measurements are capable of extracting some properties that regard complexity itself, but none of them are enough to characterize all the complex traits of a system. Mono- and multi-fractal measurements [8,9], irreversibility estimations [10,11], symbolic methods [12,13], network analysis [14,15], as well as entropy-based approaches [16,17] have been proposed to infer the system complexity.

Multiscale entropy (MSE) is an important example of an approach that has been shown to be quite robust and consistent to characterize the system complexity from HRV time series. Like many other approaches, it has some limitations depending on the situation, and improvements or refinements have been proposed since MSE has emerged [18]. For example, the entropy estimator used in MSE (sample entropy) can be replaced by other estimators, such as permutation entropy [19], fuzzy entropy [20], distribution entropy [21], dispersion entropy [22], Rényi entropy [23] and bubble entropy [24], among others. Some other entropy-based proposals, such as entropy of entropy [25] and multiscale SDiff${}_{q}$ (a measure of entropic differences) [26], are markedly different from the MSE original framework, although notably inspired by MSE.

Mild intensity aerobic exercise has been shown to improve several systemic functions and prepare the organism for sudden changes in the body. Experimental models using physical training have demonstrated that gaining physical conditioning, before an induced pathology, can reduce the disturbances caused by the disease [27,28]. In other words, physical conditioning seems to increase the system physiological complexity level. However, controversial findings have been reported about complexity and exercise, and scarce studies applied multiscale complexity approaches to identify how the aerobic training can increase the complexity in healthy subjects [29,30,31,32,33].

In the present study, we applied MSE and two other complexity measurements derived from MSE, namely multiscale dispersion entropy and multiscale SDiff${}_{q}$, to quantify the increase of complexity with physical training in experimental models of healthy rats. Results show that all measurements point to the same direction, but significant findings were obtained only with multiscale SDiff${}_{q}$.

## 2. Materials and Methods

### 2.1. Experimental Protocol

Male Wistar rats (210 g on average) were obtained from the Animal Care Facility at the Campus of Ribeirão Preto of the University of São Paulo. The animals’ usage was according to the Ethical Principles in Animal Research adopted by the National Council for the Control of Animal Experimentation, approved by the Local Animal Ethical Committee from the School of Medicine of Ribeirão Preto of the University of São Paulo.

The study divided animals into trained (n=13) and sedentary groups (n=12). Since animals could have distinct initial physical conditioning, they were individually tested for maximum velocity ($V_{max}$). For the $V_{max}$ test, the animals were placed on a treadmill, with no inclination, and the speed was increased in steps of 3 m/s every 3 min. The stage where the animal fatigued, as well as the time spent on the incomplete stage were noted to calculate the $V_{max}$ of each rat [34].

The trained group underwent a physical training protocol on the treadmill with no inclination for 9 consecutive weeks, 5 days per week. The training protocol consisted of a medium intensity training that initiated at 50% of $V_{max}$ for 20 min and ended, at the ninth week, at 70% of $V_{max}$ for 60 min (Adapted from [35]). At the fifth week, the trained group underwent a new $V_{max}$ test to adjust the training protocol as some animals acquire physical conditioning quicker than others. The sedentary group followed the same protocol, but the treadmill was kept off.

### 2.2. Data Acquisition and Processing

Two to three days after the end of the physical training protocol, rats were anesthetized with a mixture of ketamine and xylazine (50 and 10 mg/kg, ip) and implanted with subcutaneous electrodes for ECG recordings. Two days after surgery, with the animals conscious and under free movement conditions, the electrodes were connected to a bioelectric amplifier (Animal BioAmp FE136, ADInstruments, Bella Vista, Australia), and ECG recordings were acquired (2 kHz) by an IBM/PC coupled to an analog-to-digital interface (ML866 PowerLab 4/30, ADInstruments, Bella Vista, Australia).

ECG was recorded during one hour, so that multiscale measurements could be confidently estimated from HRV series. ECG recordings were processed using computer software (LabChart Pro, ADInstruments, Bella Vista, Australia) that creates HRV series as the sequence of R-R intervals, i.e., the time interval between adjacent R waves. All ECG recordings were carefully inspected, and missing beat detections and artifacts were manually corrected. HRV series are 20,000 beats in length, on average.

### 2.3. Multiscale Sample Entropy

Multiscale sample entropy (MSE) is a widely-known procedure to quantify the irregularity of time series within a time-scale range [36,37]. The MSE algorithm consists of creating multiple scaled versions of the original time series and calculating sample entropy (SampEn) from each scaled time series.

Consider a time series given by u(1),u(2),…,u(N). Let $x_{m}\left(i\right)$ be the set of consecutive samples in *u* from *i* to i+m−1, i.e., $x_{m}\left(i\right)=\left[u\left(i\right),u\left(i+1\right),u\left(i+2\right),\ldots ,u\left(i+m-1\right)\right]$. Thus, SampEn is defined as [38]:

(1) $$SampEn\left(m,r,N\right)=-\ln\frac{U^{m+1}\left(r\right)}{U^{m}\left(r\right)}$$

where:

(2) $$U^{m}\left(r\right)=\frac{1}{N-m}\sum_{i=1}^{N-m}U_{i}^{m}$$

(3) $$U_{i}^{m}=\frac{\left[\#\;\mathrm{of}\;x_{m}\;|\;d\left[x_{m}\left(i\right),x_{m}\left(j\right)\right]\leq r\right]}{N-m-1}$$

and:

(4) $$U^{m+1}\left(r\right)=\frac{1}{N-m}\sum_{i=1}^{N-m}U_{i}^{m+1}$$

(5) $$U_{i}^{m+1}=\frac{\left[\#\;\mathrm{of}\;x_{m+1}\;|\;d\left[x_{m+1}\left(i\right),x_{m+1}\left(j\right)\right]\leq r\right]}{N-m-1}.$$

The distance function *d* is given by:

(6) $$d\left[x_{m}\left(i\right),x_{m}\left(j\right)\right]=\max_{k=1,\ldots ,m}(|u\left(i+k-1\right)-u\left(j+k-1\right)|).$$

In Equations (3) and (5), 1≤j≤N−m, j≠i. In SampEn equations, *m* is the pattern length or embedding dimension and *r* is the tolerance factor assumed for similarity between samples.

To estimate MSE, multiple scaled versions of *u* are created by a coarse-graining procedure, where each element *j* in a τ-scaled series is defined by:

(7) $$u^{\tau }\left(j\right)=\frac{1}{\tau }\sum_{i=(j-1)\tau +1}^{j\tau }u\left(i\right),\;1\leq j\leq N/\tau .$$

Next, SampEn is calculated from each scaled time series $u^{\tau }$, resulting in a curve of entropy versus scale. It is worth noting that the higher the time scale (τ), the slower the dynamics that the scaled time series is representing. Importantly, the tolerance factor (*r*) of SampEn is kept fixed for all time scales (τ) in MSE.

In the present study, we calculated MSE with the most widely-used parameter setting, i.e., m=2 and r=15% of the original time series standard deviation. The maximum scale calculated was τ=20.

### 2.4. Multiscale Dispersion Entropy

Multiscale dispersion entropy (MDE) is similar to MSE and also quantifies the complexity of time series [39]. However, instead of calculating SampEn for each scaled time series, dispersion entropy (DispEn) is used to estimate irregularity.

Consider the same time series given before (*u*). First, *u* is filtered by a normal cumulative distribution function (NCDF) with mean μ and standard deviation σ, resulting in a filtered time series $u_{f}$, which ranges from 0 to 1. This procedure is intended to better treat outliers. Next, $u_{f}$ is mapped into *c* classes (1 to *c*), according to $z^{c}\left(j\right)=\mathrm{round}\left(c*u_{f}\left(j\right)+0.5\right)$, a function that linearly maps the range [0,1] to [1,c].

Now, let $y_{m}\left(i\right)$ be the set of consecutive samples in $z^{c}$ from *i* to i+m−1, i.e., $y_{m}\left(i\right)=\left[z^{c}\left(i\right),z^{c}\left(i\;+\;1\right),z^{c}\left(i+2\right),\ldots ,z^{c}\left(i+m-1\right)\right]$, i=1,2,…,N−m+1. Each vector $y_{m}\left(i\right)$ represents a dispersion pattern. Considering that each value in $y_{m}$ can assume one of the *c* possible classes, there will be $c^{m}$ potential dispersion patterns.

The probability of occurrence of each dispersion pattern $y_{m}\left(i\right)$ in $z^{c}$ can be calculated as the number of times the pattern $y_{m}\left(i\right)$ appears on $z^{c}$, divided by the total number of patterns in $z^{c}$ (i.e., N−m+1). This procedure will result in a probability distribution for all possible dispersion patterns, $p[y_{m}\left(i\right)]$. Finally, the DispEn is defined as the Shannon entropy of $p[y_{m}\left(i\right)]$ [22]:

(8) $$DispEn\left(m,c\right)=-\sum_{i=1}^{c^{m}}p\left[y_{m}\left(i\right)\right]\;\log\left(p\left[y_{m}\left(i\right)\right]\right)$$

MDE uses the same coarse-graining procedure of MSE. Thus, MDE estimation consists of the creation of scaled versions of the original time series using Equation (7) and the calculation of DispEn from each scaled time series. However, the NCDF function applied to each scaled version is the same as that applied to the first scale, i.e., the original time series. This procedure has a similar effect of keeping *r* fixed at all time scales in MSE and can be achieved choosing the same μ and σ of the NCDF function at all scales.

Parameters of MDE were set as m=2, c=6 and maximum time scale τ=20. NCDF was generated with μ and σ as the mean and standard deviation of the original time series, respectively.

### 2.5. Multiscale SDiff${}_{q}$

An alternative proposal for multiscale complexity measurement is the multiscale SDiff${}_{q}$ analysis [26]. Although still inspired by MSE in the sense of multiscale analysis, multiscale SDiff${}_{q}$ do not use the entropy values over scales directly to characterize complexity. Instead, differences of entropy between the time series and its uncorrelated version, i.e., surrogate data, are used to represent the complexity. The difference of entropy is evaluated for a range of *q*-values, which is a parameter derived from nonadditive mechanical statistics [40,41]. The so-called nonadditive *q*-entropy has three regimes, namely classic additive when q=1, sub-additive when q>1 and super-additive when q<1.

SDiff${}_{q}$ accounts for the difference between the SampEn${}_{q}$ of a given time series and the mean SampEn${}_{q}$ of a set of surrogate series. SampEn${}_{q}$ is a generalization of SampEn inspired by nonadditive statistics, which introduces the nonadditive parameter *q* to SampEn. Its equation is given by [42]:

(9) $$SampEn_{q}\left(m,r,N\right)=\log_{q}U^{m}\left(r\right)-\log_{q}U^{m+1}\left(r\right)$$

where log${}_{q}$ is defined as [43]:

(10) $$\log_{q}\left(x\right)=\frac{x^{1-q}-1}{1-q},\;\left[x\in R_{+}^{*};q\in R;\log_{1}\left(x\right)=\log\left(x\right)\right]$$

and ${\left[Z\right]}_{+}=\max\left\{Z,0\right\}$. The definitions of $U^{m}\left(r\right)$ and $U^{m+1}\left(r\right)$ are the same as presented in Equations (2) and (4) for SampEn.

To calculate SDiff${}_{q}$, one has to follow the steps:

- From a given time series *u*, *S* surrogate series are generated from *u*. The surrogate series is obtained by simply shuffling *u* [44];
- Next, values $A=U^{m}\left(r\right)$ and $B=U^{m+1}\left(r\right)$ are calculated from *u*;
- Values of $U^{m}\left(r\right)$ and $U^{m+1}\left(r\right)$ are also calculated from each surrogate instance, obtaining their mean values $C=\bar{U^{m}\left(r\right)}$ and $D=\bar{U^{m+1}\left(r\right)}$;
- Finally, SDiff${}_{q}$ is defined by Equation (11) below:
  (11) $$\begin{array}{ccc} {\mathrm{SDiff}}_{q} & = & \log_{q}\left(A\right)-\log_{q}\left(B\right)-\left[\log_{q}\left(C\right)-\log_{q}\left(D\right)\right]\\ & = & \log_{q}\left(A\right)+\log_{q}\left(D\right)-\log_{q}\left(B\right)-\log_{q}\left(C\right). \end{array}$$

Both SampEn${}_{q}$ and SDiff${}_{q}$ are parametrized in *q* so that they represent a curve of entropy, or entropy difference, as a function of *q*. From SDiff${}_{q}$ curves, three attributes (*q*-attributes) are obtained to characterize the time series dynamics, namely SDiff${}_{q_{max}}$, $q_{max}$ and $q_{zero}$. The SDiff${}_{q_{max}}$ represents the maximum value for SDiff${}_{q}$ in the range of *q*. The $q_{max}$ and $q_{zero}$ represent the *q*-value where SDiff${}_{q}$ finds its maximum and zero values, respectively. $q_{max}$ is the *q* parameter that gives the largest entropic separation between the actual time series and its surrogate versions, whereas $q_{zero}$ is the *q* parameter where original and shuffled dynamics have the same entropy. For more details on the calculation of *q*-attributes, please refer to [26,42].

The extension of SDiff${}_{q}$ to a multiscale measurement is straightforward. Scaled versions of the original time series are created using the same coarse-graining procedure of MSE, given by Equation (7). Then, for each scaled time series, the SDiff${}_{q}$ curve is calculated and *q*-attributes are obtained, so that it ends up with multiscale *q*-attributes.

Multiscale SDiff${}_{q}$ parameters were set with the same values chosen for MSE, i.e., m=2, r=0.15 and maximum time scale τ=20. The number of surrogate instances generated for each time scale was S=20, and the nonadditive *q* parameter ranged from −2 to 2 to estimate the *q*-attributes.

It is worth emphasizing the fact that *q*-attributes represent the SDiff${}_{q}$ behavior. Furthermore, the *q* parameter comes with the power law equation proposed for nonadditive entropy (*q*-entropy) [40,43]. Therefore, one can say that $q_{max}$ and $q_{zero}$ indicate where this power law results in maximum entropy differences regarding surrogates and where this difference is null (zero-crossing), respectively.

### 2.6. Statistical Analysis

We assessed mean MSE, MDE and multiscale *q*-attributes values in two range segments: short (1 to 5) and long (6 to 20) time scales. Those variables were checked for normality by the Shapiro–Wilk test. Differences between trained and sedentary groups were verified by Student’s *t*-test or the Mann–Whitney rank sum test when required. Significance was assumed when *p* < 0.05.

## 3. Results

**Figure 1:**
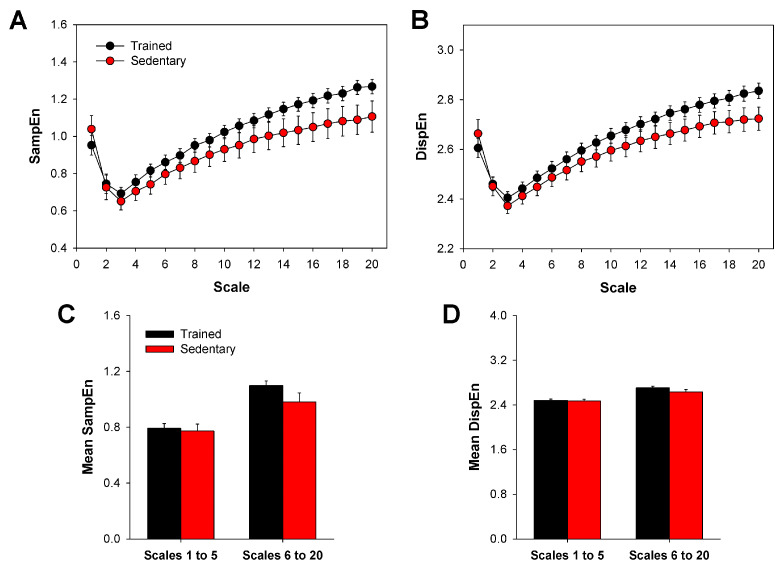
MSE or MDE did not detect differences between HRV complexity from trained and sedentary rats. Curve profiles are presented for MSE (**A**) and MDE (**B**), obtained from trained and sedentary groups. Bar graphs show mean entropy values obtained from MSE (**C**) and MDE (**D**) curves, grouped by short (1 to 5) and long (6 to 20) time scales. MSE: multiscale sample entropy; MDE: multiscale dispersion entropy; SampEn: sample entropy; DispEn: dispersion entropy; HRV: heart rate variability. Bars represent the mean ± standard error.

The curve profiles of MSE and MDE were very similar for both trained and sedentary rats (Figure 1A,B). Likewise, no difference was found between the groups in the mean values of MSE and MDE grouped by short (1 to 5) and long (6 to 20) time scales (Figure 1C,D), although for higher scales, there was a tendency of increasing differences among groups (Figure 1A,B).

**Figure 2:**
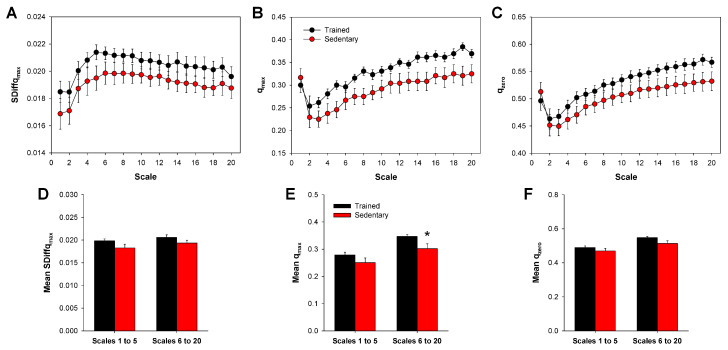
Multiscale *q*-attributes calculated from HRV series of trained and sedentary rats. Curve profiles are presented for SDiff${}_{q_{max}}$ (**A**), $q_{max}$ (**B**) and $q_{zero}$ (**C**), obtained from trained and sedentary rats. Bar graphs show mean *q*-attributes values, obtained from SDiff${}_{q_{max}}$ (**D**), $q_{max}$ (**E**) and $q_{zero}$ (**F**), grouped by short (1 to 5) and long (6 to 20) time scales. SDiff${}_{q_{max}}$: maximal SDiff${}_{q}$; $q_{max}$: *q* value where SDiff${}_{q}$ is maximal; $q_{zero}$: *q* value where SDiff${}_{q}$ is zero; HRV: heart rate variability. Bars represent the mean ± standard error. * *p* < 0.05 when compared to the trained group.

The curve profiles of $q_{max}$ and $q_{zero}$ were very similar to each other (Figure 2B,C), which in turn were also very similar to MSE and MDE (Figure 1A,B), regardless of the experimental group. On the other hand, those curves are entirely different from the profile of SDiff${}_{q_{max}}$ (Figure 2A). For $q_{max}$ and $q_{zero}$, the curve values decrease for the first two or three scales; after that, they start to increase (Figure 2B,C). However, in the case of SDiff${}_{q_{max}}$, values increase for, approximately, the first six scales, and then, the values are virtually stable (Figure 2A). A significant difference was found between trained and sedentary rats in the mean $q_{max}$ at long time scales (6–20) (Figure 2E). No difference was observed among groups in the mean SDiff${}_{q_{max}}$ (Figure 2D) or mean $q_{zero}$ (Figure 2F).

## 4. Discussion

The characterization of system physiological complexity from a univariate variable, such as HRV, is a hard task. Previous studies have reported on MSE as a powerful tool to assess the complexity of HRV [37,45,46,47,48]. Many studies have proposed and evaluated modifications in MSE, given its success in characterizing complex dynamics. Some of them are based on the replacement of sample entropy by another entropy measurement, such as MDE, attempting to improve the accuracy of MSE in specific situations. In the present study, we applied MSE and MDE to account for the complexity changes due to physical training in rats. However, neither MSE nor MDE were able to detect any difference between HRV complexity from trained and sedentary rats.

On the other hand, multiscale SDiff${}_{q}$ is a recent proposal of complexity measurements (*q*-attributes), inspired by MSE, but with a different theoretical background. It relies on nonadditive statistics and uses the difference of *q*-entropy between the actual and surrogate HRV time series to characterize the complexity. Interestingly, from all the multiscale measurements studied, only $q_{max}$ was able to distinguish the complexity of HRV between trained and sedentary animals. Moreover, the difference was found only at long time scales (6 to 20). Recent studies have pointed out that short time scales of MSE are more associated with the vagal control of HRV, whereas long time scales seem to be more related (although not exclusively) to the sympathetic control of HRV [46,49], reinforcing the existence of long-term memory in the components of the autonomic nervous system. Extending this interpretation to SDiff${}_{q}$, one could say that the difference between sedentary and trained HRV is more related to differences in the sympathetic control. This seems a reasonable assumption, given that (1) *q*-attributes use the same coarse-graining procedure of MSE to create the scaled time series and (2) physical training promotes, among other benefits, a lower sympathetic activity and modulation [50,51].

Even though there is a significant difference in $q_{max}$ between trained and sedentary groups, the difference is not huge. An interesting question to ask is: how much is changed in the physiological complexity with physical training? Another question would be: how do the interactions between physiological systems change in a physically trained animal? One has to bear in mind that all those multiscale measurements represent a general view of the system function. In other words, those complexity measurements extract the overall complexity of the system, which is the result of several mechanisms contributing to the homeostasis. Considering that the sedentary animals are healthy, a tremendous increase would not be expected in the complexity after physical training, given that most of the regulatory mechanisms are supposed to be already working at a high complexity level. Therefore, results suggest that systemic changes induced by physical training increase the system complexity to a slightly higher level.

The ability of those multiscale measurements to quantify the overall system complexity of HRV is a distinguishing feature. Many classical HRV indices seek to extract information related to the sympathetic or vagal autonomic modulation, not to mention that they are all linear models. Those indices are usually very sensitive to the environment and behavioral conditions and cannot represent the physiological complexity [4]. For example, during one hour of ECG recording, the rat may explore, sleep, groom, dig and other typical rat behaviors. All those situations will change the autonomic balance, and it is difficult to say what is the real sympathetic and vagal modulation of the rat during the whole one-hour period. On the other hand, applying the multiscale complexity measurements during the whole period, it was possible to identify that the dynamics of HRV has higher complexity in the trained rat compared to the sedentary one, even though the rat can change its physiological state several times during the recording. It is worth noting that all multiscale approaches were also applied to differential HRV series, but no difference was found between trained and sedentary animals, for any measurement [52].

The classical concept of entropy, e.g., SampEn and DispEn, relies on the quantification of the irregularity of a given series. The more irregular (unpredictable) the series, the higher the entropy. Thus, the entropy of any series is supposed to be lower than the entropy of its shuffled version (surrogate), even though the correlation properties of the dynamics were broken when samples are shuffled. However, with *q*-entropy, it is possible to achieve the same entropy values for both situations ($q_{zero}$). Therefore, if we consider the classical entropy (q=1), surrogate data will always be assigned to a higher entropy value, but if we consider *q* near 0.5 (super-additive), the two dynamics will be assigned the same *q*-sample entropy. More interestingly, there are some values of *q* where the actual dynamics is assigned higher entropy regarding its surrogate (also for super-additive *q*). Hence, $q_{max}$ can be interpreted as the nonadditive parameter that maximizes the complex properties present in the actual dynamics.

In summary, results with multiscale SDiff${}_{q}$ confirmed previous findings that $q_{max}$ and $q_{zero}$ provide similar, although not equivalent information, which is quite different from SDiff${}_{q_{max}}$. Furthermore, MSE, MDE, $q_{max}$ and $q_{zero}$ presented very similar curve profiles, despite their different theoretical definitions, and $q_{max}$ was the only measurement that detected differences in the physiological complexity after physical training. There is no doubt that MSE represents a relevant tool for complexity analysis. This study reinforces that multiscale SDiff${}_{q}$ is an alternative tool for characterizing the complexity of HRV time series, which can add information in some situations where MSE is not accurate enough. Multiscale SDiff${}_{q}$ could also be used to help to characterize the complexity of HRV time series in different pathophysiological conditions, as well as in situations where the signal source is other than HRV.
